# Supplementary material for: An HFman Probe-Based Multiplex Reverse Transcription Loop-Mediated Isothermal Amplification Assay for Simultaneous Detection of Hantaan and Seoul Viruses
Source: Diagnostics (Basel). 2022 Aug 10;12(8):1925. doi: 10.3390/diagnostics12081925 (PMC9406646; doi:10.3390/diagnostics12081925)

Supplementary Materials

Table S1. Primers used for RT-LAMP assay.

Primer sets	Primer name	Sequence (5′-3′)	Length (nt)	Genomic location (nt)
H1	F3	GTTGACAACAAGGGGGAG	18	456-655
	B3	GGTAGAGCCCACAGACTG	18	
	FIP	CGTTAACATCCTCGAACGAGCT-CAAGGATAATAAAGGGACCCG	43	
	BIP	CCGGAAACCAAAACATCTTTACG-TCTACCAGGTGTAATCTCTTCT	45	
	LB	TGTCCTTGCCAAATGCACAG	20	
H2	F3	CCAGTTATGAGTGTAATTGGTT	22	688-891
	B3	GCGTATAGCCTTTGACTCC	19	
	FIP	CTGGAAGAAGCTTGCAAGGTT-CATTAGCAAAGGACTGGAGT	41	
	BIP	GCAGCAGTTAGCCTCCTTGG-ATATTGCCTAATGCCACTTG	40	
	LB	GGTCCTGCAACAAACAGGGACT	22	
H3	F3	AGACTCCCTAAAGAGCTACT	20	8-199
	B3	GGATAGATACTGCAACGCC	19	
	FIP	CACTAATTGACCCTCATGGGCAT-AACAACGATGGCAACTATGG	43	
	BIP	GAAGGTGAGGGATGCAGAAAAA-AGTTAATGTTCTCTTGTTCAACTC	46	
H4	F3	GTTATGGTAATGTGCTGGATT	21	326-525
	B3	GCTATCATCCTTAAATCGAATCC	23	
	FIP	AGACGATGATGCTCAGCCAG-ACCATTTGGATATTGATGAACCTA	44	
	BIP	TCTTACATCCTTTGTCGTCCCG-ATCCTTGGTAGTTTGCCTC	41	
	LB	CTCTGTATATGTTGACAACAAGGGG	25	
S1	F3	AGCCCTGTCATGAGTGTAGT	20	690-897
	B3	CCTGAGGGCTTGAAATTCCT	20	
	FIP	GAACTTGCAGGGTGCGCCAA-GCACTGGCAAAAGACTGGA	40	
	BIP	GGAGTCTCCCATTGCCGGGA-AGTGCACCTTGTCTCTGTCT	40	
	LB	TCTGGAAGTCCTGTGAATCGTGA	23	
S2	F3	AGCCCTGTCATGAGTGTAGT	20	690-897
	B3	CCTGAGGGCTTGAAATTCCT	20	
	FIP	GAACTTGCAGGGTGCGCCAA-TGGGTTTTTGGCACTGGC	38	
	BIP	ATGGCGGAGTCTCCCATTGC-AGTGCACCTTGTCTCTGTCT	40	
	LF	GCCATTCTTCAATTCTAGATGTCCA	25	
	LB	GGGAGYCTATCTGGRAGTCCT	21	
S3	F3	TGGGCTATATCCTGCACAGA	20	650-862
	B3	CAAGTGCACCTTGTCTCTGT	20	
	FIP	TCCAGTCTTTTGCCAGTGCCAA-AGGCAAGGAACATGGTAAGC	42	
	BIP	CTTGGCGCACCCTGCAAGTT-CACGATTCACAGGATTCCCA	40	
	LF	ACCCAACTACACTCATGACAGG	22	
	LB	TGGCGGAGTCTCCCATTGC	19	
S4	F3	TGGGCTATATCCTGCACAGA	20	650-882
	B3	TTCCTTTGGCTCCATTCCTG	20	
	FIP	GTGCGCCAAGCCATTCTTCAAT-AGCCCTGTCATGAGTGTAGT	42	
	BIP	GGAGTCTCCCATTGCCGGGA-CAAGTGCACCTTGTCTCTGT	40	
	LF	TGCCAGTGCCAAAAACCCA	19	

RT-LAMP, reverse transcription loop-mediated isothermal amplification.

Supplementary Materials figure legends

Figure S1 Screen optimal primers for HTNV and SEOV. Different colors represent different primer pair sets. The number primer set-1, 2, 3, and 4 means the 4 pairs of primers designed and tested for HTNV and SEOV in the RT-LAMP assay.

Figure S2 The primer regions of HTNV and SEOV. A total of HTNV (326 sequences) and SEOV (400 sequences) were downloaded from GenBank on June 16, 2021. The black small dots indicate identical bases to the topmost sequence. The numbers in the parenthesis show the number of identical sequences.

Figure S1

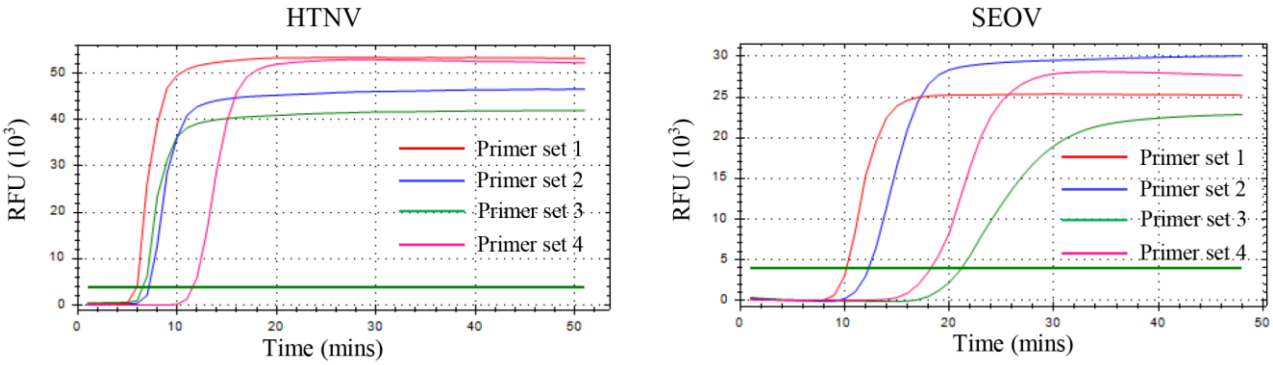

Figure S2

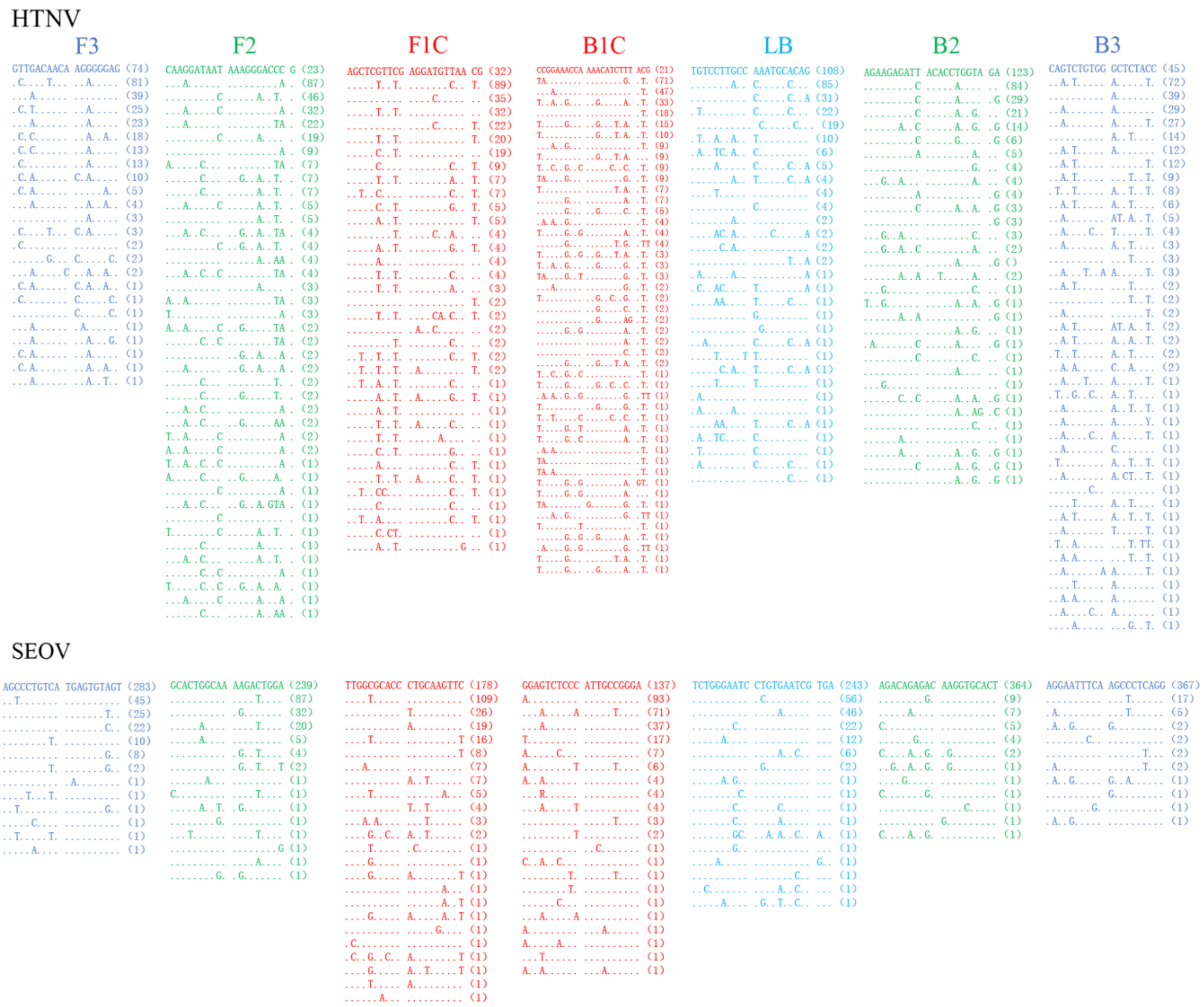

Supplement: Supplementary file 1 [file diagnostics-12-01925-s001.zip › diagnostics-1836368-supplementary.pdf]
